# Petrophysical Analyses of Rock Construction Materials from a Roman Rural Settlement in Podšilo Bay on Rab Island (North-East Adriatic, Croatia)

**DOI:** 10.3390/ma17020359

**Published:** 2024-01-11

**Authors:** Jerzy Trzciński, Emilia Wójcik, Kamil Kiełbasiński, Paweł Łukaszewski, Małgorzata Zaremba, Łukasz Kaczmarek, Robert Dziedziczak, Jakub Kotowski, Ana Konestra, Fabian Welc, Tomasz Wejrzanowski, Jakub Jaroszewicz

**Affiliations:** 1 Institute of Archaeology, Cardinal Stefan Wyszyński University in Warsaw, Wóycickiego 1/3, 01-938 Warszawa, Poland; jerzy.trzcinski@uw.edu.pl (J.T.); malgorzata.zaremba91@gmail.com (M.Z.); f.welc@uksw.edu.pl (F.W.); 2 Faculty of Geology, University of Warsaw, Żwirki i Wigury 93, 02-089 Warszawa, Poland; k.kielbasinski@uw.edu.pl (K.K.); pawel.lukaszewski@uw.edu.pl (P.Ł.); rdziedzi@uw.edu.pl (R.D.); j.kotowski@uw.edu.pl (J.K.); 3 Faculty of Building Services, Hydro and Environmental Engineering, Warsaw University of Technology, Nowowiejska 20, 00-653 Warszawa, Poland; lukasz.kaczmarek@pw.edu.pl; 4 Institute of Archaeology, Jurjevska ulica 15, 10000 Zagreb, Croatia; ana.konestra@gmail.com; 5 Faculty of Materials Science and Engineering, Warsaw University of Technology, Wołoska 141,02-507 Warsaw, Poland

**Keywords:** Roman Dalmatia, heritage, ancient materials, sedimentary rocks, SEM-EDS, microtomography, mechanical properties

## Abstract

This article presents the results of petrophysical analyses of limestones and sandstones used for the construction of the wall structures of a Roman rural settlement located in Podšilo Bay on Rab Island (Croatia). An on-site analysis of the walls indicated the use of different lithotypes, which is an uncommon case in the area. So far, no petrophysical properties of the applied materials have been tested, and their provenance has not been specified. The aim of this research was to determine their usability as construction materials in an attempt to determine the possible reasons behind the usage of multiple lithotypes and their suitability as building materials. The following procedure was used to address these issues: (1) determination of the petrographic characteristics of the rocks, (2) performance of tests to characterise the mechanical properties in a complex stress state of uniaxial tension followed by uniaxial and triaxial compression, and, finally, (3) determination of the internal structure of the rocks using methods based on X-ray imaging. Multi-proxy characteristics of the materials including numerous observations and methods were performed: optical microscopy used to characterise rock petrography and mineralogy, scanning electron microscopy (SEM) coupled with EDS, as well as grinding tests; furthermore, mechanical properties were determined on cylindrical samples in accordance with European standards. X-ray microtomography using the XμCT method enabled microscopic observations and determination of the orientation of discontinuities and the rock structure. The performed analyses allowed us to distinguish three lithological types of sandstone and two types of limestone among the examined stone blocks. Stone blocks of fine- and medium-grained sandstone with carbonate binders, as well as sparitic limestone and mudstone with calcite veins, were used to build the studied structures. The analysed blocks showed traces of partial edge processing. Despite the defects in the material structure identified using XμCT, all the types of rock were characterised by high or very high strength. High values of longitudinal wave velocity confirmed the good quality of the material. These results contribute to a better understanding of the construction process and the related technological choices, and they provide the first dataset which can be used for the reconstruction of the building’s original appearance in the future.

## 1. Introduction

**Figure 1:**
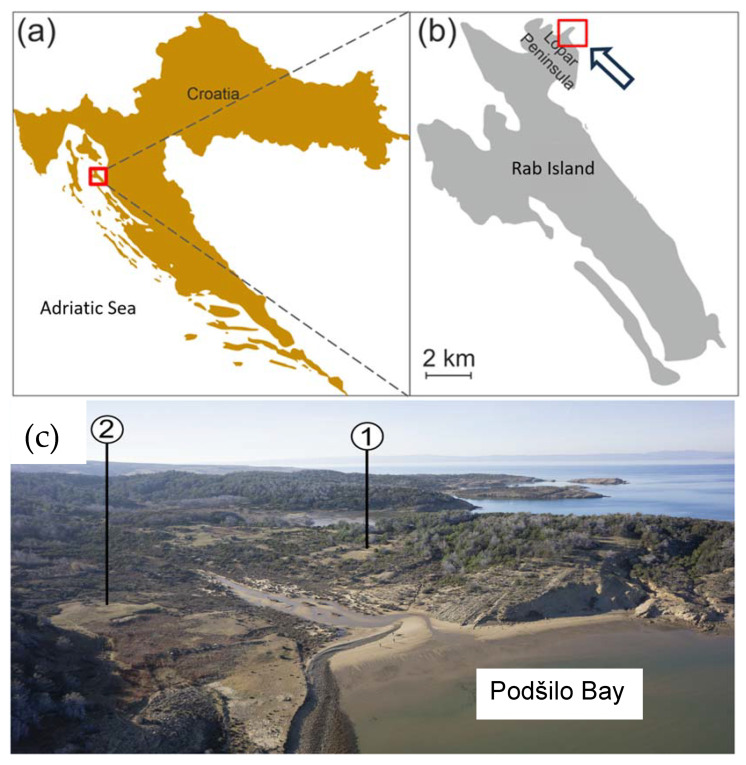
Location of the studied archaeological site. (**a**) Map of Croatia with the location of Rab Island marked with a red square; (**b**) map of Rab Island and the Lopar Peninsula. The red square indicates the location of the archaeological site in Podšilo Bay; (**c**) view of Podšilo Bay from the east, with the location of the excavation trenches (1. Beli grad; 2. Podkućine).

The Rab Island is located in the Kvarner Gulf, the northernmost inlet of the Adriatic Sea, just off the coasts of the Velebit mountain range (Figure 1a,b). The island was first mentioned as Arva/Arba by Greek and Roman geographers [1,2]. During the Iron Age, the region was inhabited by local communities possibly belonging to the wider Liburnian cultural group. With the onset of Roman rule during the 1st century BCE, the region became part of the province of Illyricum, and later of the province of Dalmatia [1,3]. During that period, modes of settling and economic exploitation changed significantly. The first Roman rural settlements (“villae”) started to be constructed in the countryside and on the seashore as centres of landed estates, thus combining residential and economic functions [3,4]. Therefore, apart from residential building(s), they often contained production facilities and workshops for various craft productions. Along with new types of settlement, novel construction techniques appeared in the region. The coast and islands of Roman Dalmatia are dotted with numerous sites built in Roman tradition using stone blocks bound by lime mortar and different, sometimes locally produced, ceramic building materials, such as ceramic roofing tiles [5]. Typically, locally or regionally sourced limestone was the preferred construction material in both urban and rural settings, as testified by the structural remains of buildings but also by numerous Roman-time quarries identified along the Eastern Adriatic coast and on the adjacent islands. However, none have so far been found on Rab Island [6,7].

One such rural productive settlement has been identified in Podšilo Bay on the Lopar Peninsula in the northern part of Rab Island (Figure 1a). Based on a geophysical survey and archaeological excavations led by a Polish–Croatian team, remains of several buildings and tile kilns were located in different areas of the bay’s coastal belt and its hinterland [8,9,10,11,12]. Other finds suggest a multiphase occupation of the site, ranging most probably from the 2nd to the 6th centuries CE, and the existence of iron working at the site [13]. The two so-far excavated buildings, located, respectively, in the areas of Beli grad and Podkućine (Figure 1c), have been interpreted on the basis of GPR and excavation results [10] as a large, probably residential building consisting of an internal courtyard surrounded by wings with several rooms, and a smaller, possibly storage house or economic building.

Analyses of the discovered wall structures of both architectural complexes, in places preserved at a total height of over 1 m, have pointed out several peculiarities, particularly the use of both limestone and sandstone blocks in both the foundations and wall elevations. Sandstone has very seldom been noted as a construction material in Roman times in the region, with limestone prevailing in other sites of the Lopar Peninsula. Since 2019, a programme of geoarchaeological research has been initiated for a better understanding of the building materials used at the site, mainly stones and mortars used in masonry, as well as answering questions regarding the origin of the applied materials, organisation of the building site, and the logistical and technological choices made by both the investor and the builders, all being indicative of the necessary expertise and investment needed for such an endeavour [14,15]. Moreover, such research allows us to gain insights into the wall structures, allowing us to infer on their original appearance.

The primary objectives of the research conducted are twofold.

The first objective was to characterise in detail the petrographic properties to determine the general provenance of the used rock material. According to literature data, similar petrographic studies were initially conducted only for stone monuments, and the research was mainly focused on Neolithic axes [16]. Currently, these studies are referred to as petroarcheology in Central Europe and petromineralogy in other regions of the world [17].

The next objective was to identify the mechanical properties and potential suitability for construction. Studies on the strength of ancient masonry are more often applied to large-scale construction works of Roman public and urban buildings, e.g., [18,19,20,21], often well preserved until today; and the studies have so far been mostly confined to a few localized areas of the Roman world [20,22,23]. Thus, the obtained results provide novel data regarding rural settlements and the organisation of their building process, e.g., [24,25], as well as the first dataset for the province of Dalmatia.

**Figure 2:**
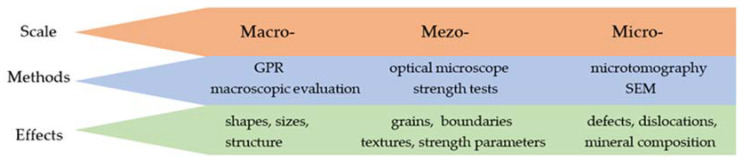
Scheme of methodological approach for the archaeological evaluation of construction materials.

These aims are achieved with a methodology that has not yet been applied to such structures in the region. In addition to the characterization of the stone materials used in the structures on Rab Island, the paper presents a previously unpublished research approach that uses imaging representative of full-scale magnifications (Figure 2).

The use of different imaging methods in the evaluation of building materials is a significant contribution to the originality of the article.

The proposed research methodology is flexible and has the following capabilities:

(1) It can be used for a variety of structures of any size and arrangement in plans, the contours of which can be recognized by geo-radar prospecting (GPR);
(2) It can be applied for the recognition of any rock material, both in terms of identification and mineral composition studies (optical microscope, SEM EDS);
(3) It allows for the planning of specialized geomechanical tests with low availability of test material (multistage stress tests preceded by detailed recognition with X-ray tomography).

The results of the research presented herein are part of the developing trend of archaeometric research, where archaeological research is assisted using methods from the sciences., e.g., geophysics and geomechanics, which are becoming integral parts of archaeological research.

## 2. Geological Setting

**Figure 3:**
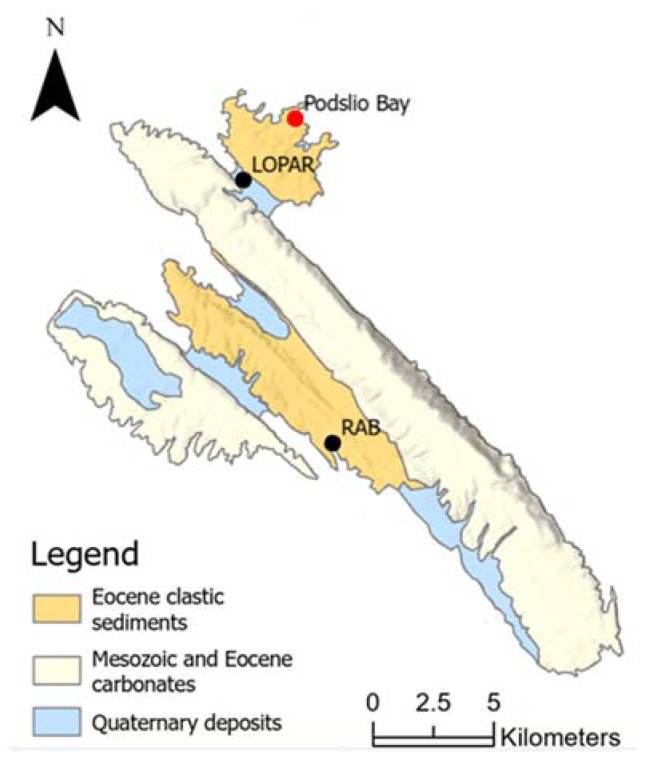
Geology of Rab Island (after [26], modified).

The geological setting of Rab Island consists of two synclines and two anticlines (Figure 3). The oldest rocks exposed on the island are Upper Cretaceous (Cenomanian—Turonian) grey-brown, bedded limestones with intercalations of dolomitic limestone, and white and yellowish, generally non-stratified, crystalline limestones. Upper Cretaceous (Turonian–Maastrichtian) strata comprise light grey and white, occasionally reddish limestones [26]. Deposits of the lower and middle Eocene consist of foraminiferal limestones (E1–E2), while those of the middle and upper Eocene comprise flysch deposits: marls (E2–E3, lower flysch) and yellowish sandstones (E2–E3, upper flysch). Flysch deposits are characterised by alternations of fine and coarse-grained sediments. Moreover, Eocene sandstones are dominated by detrital grains identified as angular to subangular quartz grains; feldspars; mica; heavy minerals, e.g., zircon; rutile; tourmaline; and other lithic fragments, e.g., pelitic, quartzite type, and quartz-feldspar aggregates. The carbonate content of the samples is 35% and 48%, and it is concentrated primarily in the matrix [27].

The youngest sediments on the island are Quaternary quartz sands of eolian and alluvial origin. The Lopar Peninsula is built of Paleogene flysch sediments, while Quaternary formations are observed in deep valleys, in the area between the Cretaceous carbonate anticline in the southwest of the island and the flysch syncline in the northeast of the peninsula (Figure 3). Quaternary strata are characterised by good sorting, rounded grains, and the absence of carbonate components [26,28].

## 3. Material and Methods

### 3.1. Material

**Figure 4:**
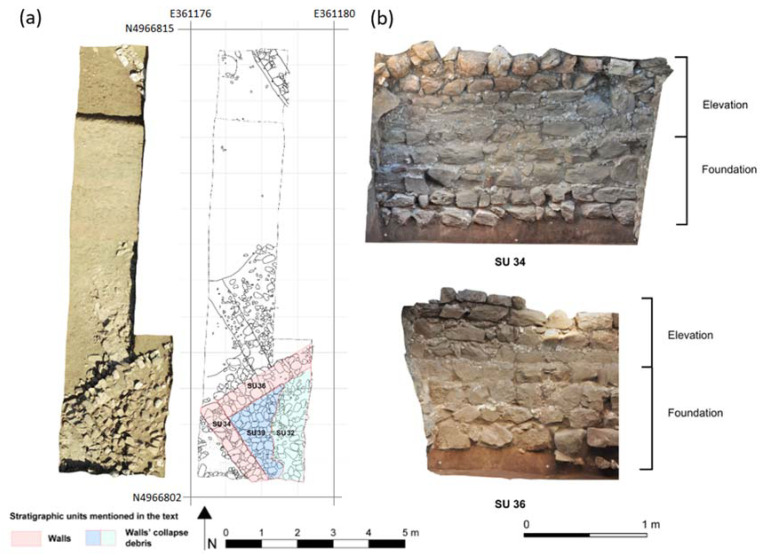
Excavation of the Roman structures at Beli grad. (**a**) Photograph and map of the trench with marked archaeological layers (drawing by K. Rabiega, modified), (**b**) cleared part of the preserved internal walls SU 34 and SU 36.

The material for petrophysical and endurance research was collected during excavation in Trench 2 in the Beli grad area of Podšilo Bay (Figure 4a,b). The trench was aimed to explore parts of the eastern wing of a building of probable residential use. Part of the stratigraphy in the southern part of Trench 2 comprised a thick layer of rubble collapsed from the adjacent walls—SU 34, 36. The foundation of these walls was mostly constructed with irregular, medium-sized blocks of sandstone and limestone, bound with lime mortar only in their upper part. The wall structure consists of two leaves built of roughly worked sandstone and limestone blocks, joined by lime mortar so that they form regular rows of various heights. The wall core consists of smaller stones bound together with mortar. Walls SU 34 and SU 36 were probably the internal walls of the building (Figure 4b).

Stone samples were collected from SU 32, SU 36, and SU 39 (Figure 4a). SU 32 constitutes the upper part of the destruction debris mixed with soil, SU 36 is the wall extending from NE to SW in the trench from which a loose stone was extracted, while SU 39 is another layer of collapsed stones that lies below SU 32, roughly mixed with soil.

### 3.2. Methods

#### 3.2.1. Polarised Light Microscopy

Thin sections of 30 micron thickness were analysed with a Delta Optical POL-1000TRF [Delta Optical, Nowe Osiny, Poland] petrographic optical microscope equipped with a DLT-Cam PRO 20 MP [Delta Optical, Nowe Osiny, Poland] digital camera. The analyses included the determination of the rock texture and structure (arrangement of particular grain fractions and pore sizes, shapes, rounding, and the distribution of mineral components), mineral identification, as well as diagenetic changes and transformations, type of matrix, relics of the original minerals, and mineralization. Magnifications of up to 50 times were used. 

#### 3.2.2. Scanning Electron Microscopy (SEM)

Backscattered electron imaging in a field emission scanning electron microscope (FE-SEM-BSE) was carried out with a ZEISS SIGMA VP [Carl Zeiss Microscopy, Cambridge, UK] field emission electron microscope. Initial phase identification was performed using the SEM-EDS method (energy-dispersive X-ray spectroscopy) using two Bruker XFlash 6|10 EDS detectors [Brucker Nano GmbH, Berlin, Germany].

The performed analyses provided the morphological properties and the elemental chemical composition of the grains occurring in the analysed samples. The analyses were performed in the following conditions: accelerating voltage—20 kV, beam current—70 mA, WD (working distance)—10 mm, and acquisition time of the analysis—100 s. (live time). Analyses of the elemental composition and imaging were conducted in a low-vacuum mode (20–40 Pa); therefore, coating the samples with a conducting layer of carbon was unnecessary.

#### 3.2.3. X-ray Microtomography

X-ray computed microtomography (XμCT) was used to analyse the internal structure of six samples, employing a rotation technique for multi-viewpoint image acquisition [29,30]. After drying, six samples were analysed using Bruker microCT [Brucker Nano GmbH, Berlin, Germany] and Avizo Fire software (version 8.1) for qualitative descriptions and numerical visualizations. Three-dimensional models aided in analysing fractures and void distribution, with spatial analyses used to calculate parameters such as porosity. While the acquired microCT images identified the crucial structural changes, components smaller than the voxel size required scanning electron microscopy (SEM). Despite SEM limitations, XμCT and SEM complement each other in the presented study.

The used XμCT setup (Zeiss Xradia Micro XCT 400 tomograph—manufactured by Carl Zeiss X-ray Microscopy, Inc., Pleasanton, CA, USA) involved an air-cooled Hamamatsu L8121-03 X-ray tube with tungsten anode-generating conical X-rays. The experiment involved a single radiograph with an 8 s. exposure time at a voltage of 120 kV and a power of 10 W. It took approximately 4 h to create the set of spatial images for a single sample using around 800 radiographs. The voxel sizes were in the range of 40–50 microns at the selected technical exposure settings, representing the smallest distinguishable structural element. Raw images obtained during scanning were converted into an 8-bit digital format with a resolution of 1024 × 1024 pixels. The reconstructed tomographic images were presented in greyscale, where the lighter areas corresponded to higher-density zones.

#### 3.2.4. Geomechanical Tests

The geomechanical properties of the rock samples were determined using basic and strength tests. The basic tests included determining the rock density, whereas the rock strength tests included unconfined, uniaxial compressive strength; indirect tensile strength; and triaxial compressive strength. Additionally, for identification purposes, non-destructive ultrasonic tests were conducted to determine the longitudinal wave velocity of the studied rocks.

The rock samples were prepared from rock monoliths; each had a diameter of about 38 mm, a slenderness ratio of about 2 for uniaxial and triaxial compressive strength tests, and a diameter of about 38 mm and a slenderness ratio of about 0.5 for tensile strength.

In order to determine the changeability of the elastic properties of the rock material, all of the samples prepared for the destructive tests were subjected to non-destructive ultrasonic tests to assess their elastic parameters. During the tests, the longitudinal wave travel time in the direction parallel to the specimen axis was measured to calculate the velocity (V_p_). The measurements were performed using the transition method with an ultrasonic flaw detector.

Uniaxial and triaxial compressive strength tests were carried out in a stiff loading machine MTS-815, produced by MTS Systems Corporation (Minneapolis, MN, USA). Tensile strength tests were performed in the Automax 50 loading machine of the Controls company (Liscate, Milan, Italy). 

##### Unconfined Uniaxial Compressive Strength Tests

On the basis of strength tests under uniaxial compression stress, the following parameters were determined:

- Uniaxial compressive strength σ_u_,
- Elastic constants: Young’s modulus E and Poisson’s ratio ν.

Uniaxial compressive strength was determined using Method D according to the ASTM D 7012-14 [31] test procedure and the ISRM recommendations [32]. During the test, load was applied continuously at a standard constant stress rate of 1 MPa/s. For samples with a diameter of about 38 mm, the load rate was 68 kN/min. This allowed for determining the value of failure load P with an accuracy of 1 kN.

Additionally, axial strain (ε_a_) and lateral strain (ε_l_) were recorded using extensometers produced by MTS. The values of volumetric strain (ε_v_) were calculated on the basis of axial strain values.

According to the standard ASTM D 7012-14 [31], experimental stress–strain curves were used to evaluate the elasticity parameters such as Young’s modulus E and Poisson’s ratio ν. The values of the Young’s modulus and the Poisson’s ratio were calculated for the interval from 25 to 50% of the maximum stress.

##### Triaxial Compressive Strength Tests

On the basis of compressive strength tests under triaxial stress conditions, the following parameters were determined:

- Differential failure stress σ = σ_1_ − σ_3_,
- Elastic constants: Young’s modulus E and Poisson’s ratio ν.

Triaxial compressive strength tests were conducted according to the [31] ASTM D-7012-14 standard, as well as the recommendations of ISRM [32], where the so-called “multiple failure test” was applied. This is one of the three types of tests recommended by the International Society for Rock Mechanics [33] for triaxial compression testing. Triaxial multiple failure tests were performed at four stages of different confining pressures for each sample. The triaxial compressive strength of the test specimen was calculated according to Method B ASTM D7012-14 [31]. Young’s modulus E and Poisson’s ratio ν were determined in the same way as in Method D of the uniaxial compression test.

Triaxial compression tests performed at four different confining pressures for rock material from a particular stone block allowed to determine the values of the angle of internal friction φ and of cohesion c, based on the failure envelope and in accordance with ASTM D7012-14 [31]. The choice of this type of testing was dictated by the need to perform uniaxial compressive strength, tensile strength, and microscopic testing within a single stone block with a size of 20/20/30 cm, resulting in a limited amount of material for triaxial testing. In addition, XμCT scanning of each specimen for triaxial testing would have been both time- and cost-consuming.

##### Indirect Tensile Strength Tests

Tensile strength was assessed using the Brazilian test method and based on a test procedure in accordance with ASTM D 3967-08 [34]. The Brazilian test involves loading a cylindrical rock sample with a compression force which is evenly distributed along the diametric line. During the tensile strength test, the load was continuously increased with a standard constant stress rate of approximately 0.15 MPa/s. For samples with a diameter of about 38 mm, this gave a load rate of 10 kN/min. During the test, failure load P was recorded with an accuracy of 0.1 kN.

## 4. Results

### 4.1. GPR Results

**Figure 5:**
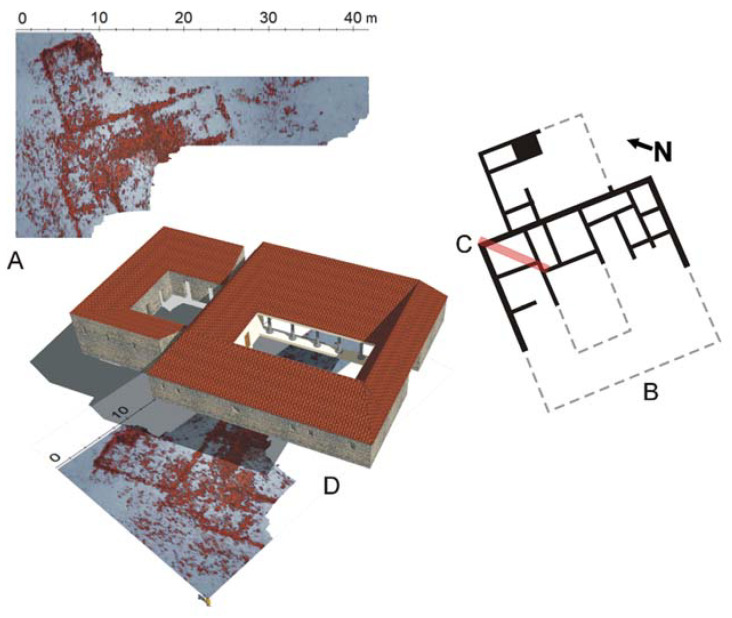
(**A**) Ground-penetrating radar prospecting results showing the remains of a villa at the depth of approximately 0.40 m. Notable is the central courtyard—atrium. (**B**) A tentative reconstruction of the villa plan on the basis of geophysical surveys and excavations. (**C**) Location of the archaeological excavation; see text for a detailed discussion. (**D**) A tentative reconstruction of the villa appearance. (Elaboration of GPR results and drawings: F. Welc).

Following the discovery of a Roman pottery kiln on the shore of the bay, conducted geophysical surveys have identified the location of the settlement’s complex structure. Based on the excavations, two structures were identified: a smaller, possibly auxiliary building on the southern slope and a larger complex on the northern slope, interpreted as the settlement’s central building (villa). As shown by GPR measurements (GPR), the structure of these buildings is located around an enclosed space [9], perhaps a courtyard, with wings divided into multiple rooms of a yet undetermined function. The surveys have provided the basis for inferring the size of the buildings on the plan (Figure 5), but, unfortunately, when applied to the buildings in their current state, they did not provide answers to either their size or the number of storeys. Most of the Roman archaeological sites in Croatia from the discussed period, including buildings, whether residential or productive structures, are usually preserved only at the foundation level. This is the main reason why it is difficult to reconstruct their original appearance.

### 4.2. Macroscopic and Microscopic Analyses

**Figure 6:**
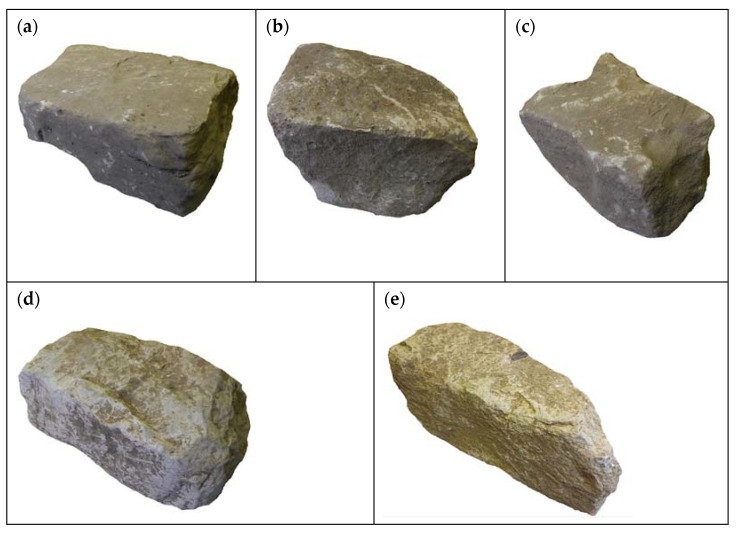
Stone blocks selected for further analysis. (**a**) Type LS_1, dark-grey fine-grained sandstone; (**b**) type LS_3, light-brown medium-grained sandstone; (**c**) type LS_6, light grey fine-grained sandstone; (**d**) type LL_7, dark grey mudstone; (**e**) type LL_10, grey sparitic limestone; LS (Lopar Sandstone), LL (Lopar Limestone).

Three lithological types of sandstones were distinguished (Figure 6). The studied stone blocks mainly had rounded edges. Macroscopically, for the fresh fracture in sample LS_1, they were dark grey with a low-intensity and short-lived reaction with 10% hydrochloric acid, in sample LS_3—light brown with an intense and long-lasting reaction with 10% hydrochloric acid, while in sample LS_6—light grey with the most intense reaction with hydrochloric acid. The reaction occurred with both the grains and the binder. Based on the grain size (Figure 6a–c), the rock types were classified as medium-grained (LS_3) and fine sandstones (LS_1, LS_6). The grain framework in sample LS_1 was loose and fine-grained. It was mainly composed of sharp-edged, poorly rounded, and well-sorted quartz. Most of the quartz grains displayed an undulatory extinction. Numerous inclusions were seen in the quartz grains. The grains were unoriented. In addition to quartz, the framework also contained feldspars (mainly K-feldspar), muscovite, ilmenite, rutile, titanite, iron oxides, a few lithoclast fragments, and chalcedony grains with spherical shapes. Sample LS_6 had a similar texture, with the grains being slightly larger and better rounded. Sample LS_3, on the other hand, was medium-grained. Its framework was more compacted, and the quartz grains are larger, sub-rounded and moderately sorted. The grain framework contained K-feldspars, lithoclast grains, sparse accessory-heavy minerals, and muscovite. 

The EDS analysis confirmed observations in the polarising microscope; it shows variations in the mineral composition of the grain skeleton of the different sandstone types (Supplementary Materials). In sample LS_1, grains of zircon, pyrite, apatite, and chromite Cr-spinel were found. In sample LS_3, mixed potassium-sodium and sodium-calcium varieties were also identified among the feldspars. In addition, zircon, alumina grains, and pyroxenes were observed. In sample LS_6, grains of iron oxides were abundant. Moreover, grains of pyrite, Cr-spinel, and biotite were also discovered.

**Figure 7:**
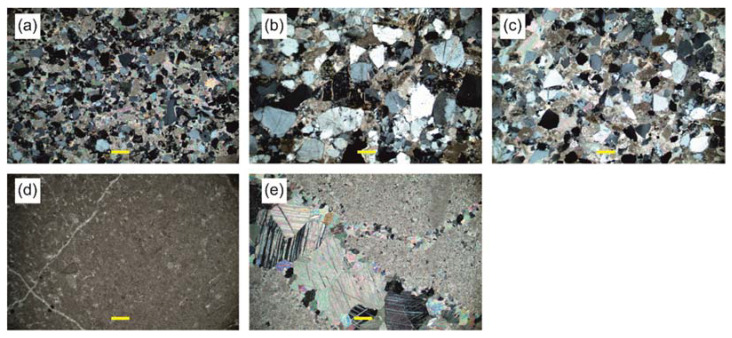
Thin-section photomicrographs with transmitted light optical microscope under crossed polarizers. Scale-bar is 200 μm (marked with a yellow line). (**a**) Type LS_1, moderately sorted, fine-grained sandstone with carbonate cement; (**b**) type LS_3, moderately sorted, medium-grained sandstone with carbonate cement; (**c**) type LS_6, well-sorted, fine- to medium-grained sandstone with carbonate cement; (**d**) type LL_7, mudstone with visible fine veins of recrystallized calcite and foraminifera; (**e**) type LL_10, fine grained limestone with veins of calcite crystals.

Macroscopically, two lithological types of limestone were distinguished (Figure 6): dark grey LL_7 with an intense but short-lived reaction with 10% hydrochloric acid, and grey LL_10 with an intense and long-lived reaction with 10% hydrochloric acid. Figure 6 clearly shows the rounded edges of the stone blocks. Optical microscopy observations show that, according to the classification by Folk [35,36], the rock types include mudstone (LL_7) and sparitic limestone (LL_10), whose matrix is intersected with veins of coarse crystalline calcium carbonate. In both cases, numerous small bivalve and foraminifera shells were observed in thin sections (Figure 7d,e). SEM images show a grey background, which, according to the chemical analysis, is composed entirely of calcium carbonate. SEM-EDS analysis revealed average oxygen contents at 28.7 and 28.5 percent and calcium contents of 70.9 and 71.5 percent for samples LL_7 and LL_10, respectively. There were trace amounts of magnesium, aluminium, silicon, and iron in sample LL_7 (Supplementary Materials).

### 4.3. X-ray Microtomography Analysis

Macroscopic observations and petrographic studies were performed by determining the characteristics of the internal structure in the analysed cylindrical sandstone and limestone samples.

**Figure 8:**
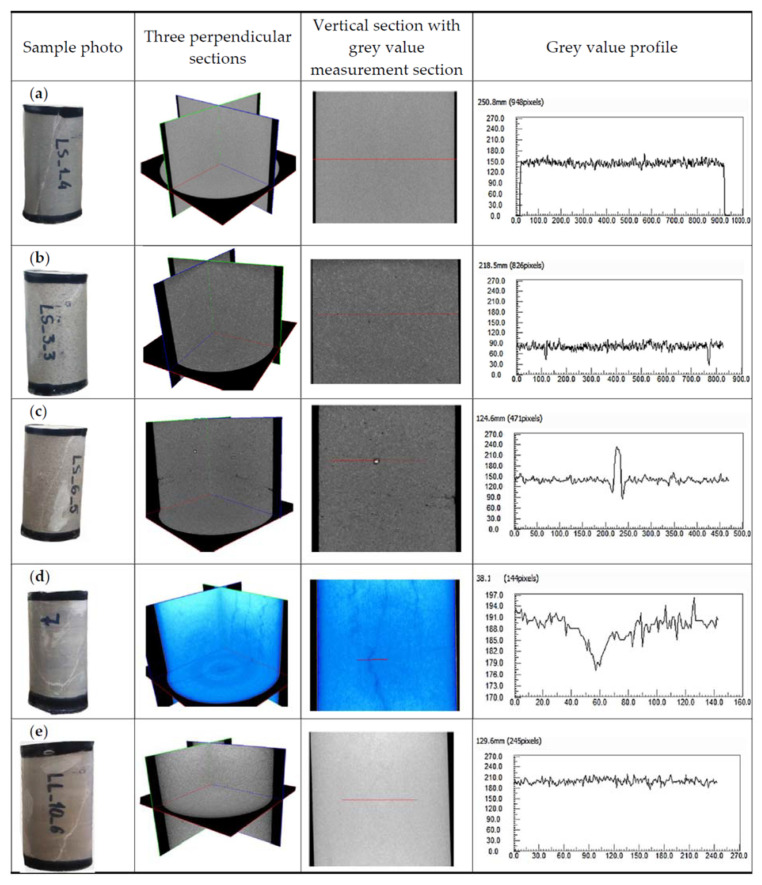
Microtomography images with grey value profiles for (**a**) type LS_1, (**b**) type LS_3, (**c**) type LS_6, (**d**) type LL_7, (**e**) type LL_10. —— section line.

An image analysis of sample LS_1 showed no structural defects (Figure 8a). The entire stone was homogeneous with regard to grain size. Due to the size of individual grains being close to the imaging resolution, it was not possible to observe their details. To complement this magnification range, a SEM analysis was used, which is very suitable for such type of study (despite the small area of recognition, it provides a detailed identification of the grain characteristics)—Figure 8a. The porosity is very low, about 0.02%, and comprises pores with equivalent diameters in the range of 1.5–124.5 μm (about 97%).

The image of sample LS_3 is very similar to that of sample LS_1. The rock has a compact structure with a low porosity of about 0.08% and with a predominance of pores with an equivalent diameter of up to 0.1 mm (about 73%). The density analysis performed in the cross-section showed very high homogeneity (Figure 8b).

The XCT scan of the last sample in the sandstone group (LS_6), except for the evenly dispersed porosity, showed the presence of small voids in the sample volume, which may affect the strength. Besides voids, the presence of grains composed of very-high-density minerals was observed as white “grains” in microtomographic images (Figure 8c).

The 3D image of limestone LL_7 reflects the internal structure of the sample, where the weakness areas (depicted with a darker colour) are identified in three perpendicular sections—they most likely represent fracture/gap areas filled with secondary mineralization. These zones of weakness are characterised by quasi-parallelism, and their course is not straight. The degree of weakening of the rock’s solid fraction may correspond to the grey value profile (related to the density value) (Figure 8d).

CT images are presented with shaded grey or black colours for low CT-values and light grey or white colours for high CT-values in all subsequent shaded grey colours. The total number of levels in these colours is 256. It is well known that this CT-value is linearly related to the material density [37]. The grey value profile in Figure 8 shows the distributions of CT-values along exemplary lines in all specimens, showing the relative density variance of the analysed materials.

Variability related to mineralization in the form of secondary crystallisation in cracks was noted in sample LL_10. Such elements may represent a favoured area of failure in the context of lower strength than the primary structure of the analysed carbonate rock. The largest number of filled cracks is located in the lower part of the sample, which, however, continues reaching the middle part of the analysed area at different angles. Hence, the average extension of the described internal structures can be found to be about 2 cm (with a dilation of about 0.2 mm). The small density variation translates into a homogeneous microtomographic image (Figure 8e). The results of the observations and XCT imaging measurements for all specimens studied are shown in Table 1. 

### 4.4. Geomechanical Properties

The lithological variability, which is clearly visible in the macroscopic analysis, is also reflected in the test results of the bulk density and ultrasonic properties. Medium bulk density values for sandstones are lower than for limestones, and the values decrease in the following sequence: LS_3 > LS_6 > LS_1 vs. LL_7 > LL_10 (Table 2).

Longitudinal ultrasonic waves spread with different velocities for the tested rocks. The medium values of the longitudinal wave velocity ranged from 3258 to 4632 m/s for sandstones and from 6335 to 6375 m/s for limestones, respectively. Changes in the velocity values maintained the trends of the bulk density variation (Table 2). The highest average values of longitudinal wave velocity were measured for both types of limestone. Twice smaller values were recorded for sandstone types LS_3 and LS6. Medium values were obtained for type LS_1 (dark grey fine-grained sandstone).

The values of the uniaxial compressive strength ranged from 87 to 140 MPa (Table 3). The highest values of uniaxial compressive strength were recorded for types LS_6 and LS_1. The highest strength values of uniaxial compression were unarguably associated with the structure of the analysed rocks. The XCT imaging results indicated their low porosity at 0.02–0.17% in the form of small pores; 41.5–207.5 μm in size, which was evenly distributed in the volume of the samples; and with no obvious discontinuity surfaces.

Medium values of uniaxial compressive strength were recorded for type LL_7, and the lowest were for types LL_10 and LS_3. Some cracks filled with secondary mineralization were observed in limestone samples LL 7 and LL 10. The XCT imaging results indicated low variability in limestone density and mineralization. Despite the fact that the cracks were completely filled, they contributed to the integrity of the rock structure, allowing for strain propagation along these structural heterogeneities.

**Figure 9:**
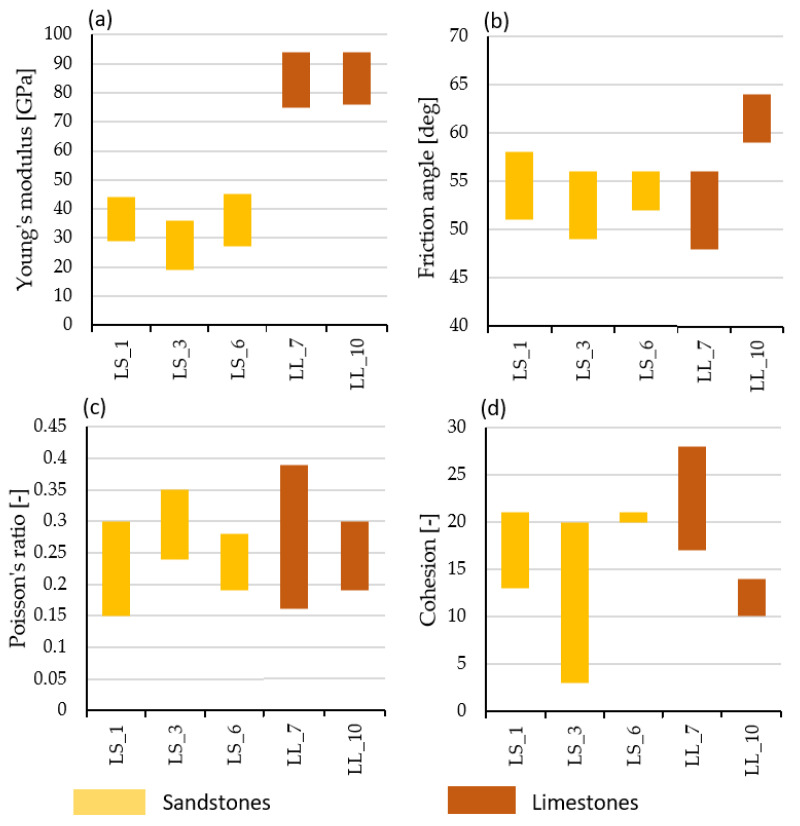
Triaxial multiple failure test results. (**a**) Young’s modulus E [GPa], (**b**) friction angle φ [deg], (**c**) Poisson’s ratio ν [-], (**d**) cohesion c [MPa].

In terms of strength, types LS_6, LS_1, and LL_7 belonged to very strong rocks, with a strength of more than 100 MPa, while types LL_10 and LS_3 belonged to strong rocks, with strength in the range from 50 to 100 MPa according to PN-EN ISO 14689-2018 [38]. A similar variation was observed in the values of indirect tensile strength, which ranged from 4 to 15 MPa (Table 3). The purpose of triaxial strength tests of the “multiple failure type” was to define the value of the strength parameters c and ϕ (Figure 9), which, in the future, are to be used to create a numerical model for the reconstruction of other walls of the examined buildings.

## 5. Discussion

Petrographic studies have shown that the Roman buildings located on Rab Island in Podšilo Bay were erected using fine- and medium-grained carbonate-bonded sandstones, mudstones, and sparitic limestones intersected with veins of coarse crystalline calcite of local origin. These raw materials are readily available near the site, and the character of the block surfaces indicates little processing.

Foraminifera observed in both blocks of limestone indicate that local Eocene limestones were used for the construction of the Roman structures. The petrographic characteristics of the sandstones also point to their local origin. The analysed stone blocks vary in size and show little edge processing, with some of them rounded, and with burnt lime mortar used to join them [11]. This suggests the use of rock fragments from nearby locations such as, for example, streams. The acquired data, coupled with further fieldwork, might, in the future, aid in determining a more precise source of the limestone blocks, and thus allow us to estimate the possible transportation distances to the building site, the related labour, and cost [39]. It should also be kept in mind that limestone was used to manufacture the lime mortar used as a binder. The raw materials chosen for construction, despite the passage of time, still have good strength parameters.

The sustainability of masonry structures depends mainly on three factors. These are the strength of the materials, the geometry of the elements (bricks) and joints used in masonry construction, the geometric arrangement of the structure, and the construction technique.

Typical mechanisms of joint failure in masonry structures according to [40] are:

- Joint tensile cracking and joint slipping—dependent on mortar strength and joint geometry,
- Unit direct tensile cracking and unit diagonal tensile cracking—mechanisms that depend on the strength of bricks or stone blocks (units),
- Masonry crushing—mechanism conditioned by the strength of the masonry as a unit.

In combination of the above-mentioned factors with the mechanisms of failure of masonry structures, we can only talk about the sustainability of masonry structures.

Given the above, among other things, it is the material characteristics such as strength that will determine not only the load-bearing characteristics of the walls, but also the method of their construction. Walls made of stone materials of different genesis (magmatic, volcanic, sedimentary, metamorphic) will significantly differ in physical and mechanical properties, and this will reflect on the way structures are formed.

Besides the stone material, an important factor affecting the strength of the masonry is the quality of the mortar. Our research was originally intended to also include mortar strength testing. However, due to the poor condition of the mortar, resulting in the inability to cut out standard samples for strength testing, this type of testing could not be continued. The poor quality of the mortar may have contributed to the failure of the investigated buildings. In addition, the buildings were located along the coast, where elevated humidity is normal. Therefore, the main factors of accelerated mortar weathering being increased moisture and temperature fluctuations provide support for this theory. However, it is not only in the properties of the mortar itself that the causes of failure of the tested structures should be considered.

The literature often presents the view that the initial parameters of the mortar bonding the stone elements determine the subsequent vulnerability of the structure to failure processes [41].

The geometry of the stone blocks themselves used in the construction of the wall is also of significant importance here. It has been proven that masonry with a random arrangement of stones bound by mortar (rubble stone masonry) has worse resistance to seismic effects than those built from worked stone blocks (coursed stone masonry) or built blocks with regular shapes without mortar (dry stone masonry) [42].

However, this factor cannot definitively determine the strength of a masonry structure, especially in structures with a random arrangement of stones. Sometimes the mechanism of interlocking stones occurs randomly, which can locally strengthen the structure, and despite using the same material and construction technique, part of the structure will be more resistant to seismic events.

The third factor, construction technology, is what we believe the ancient structures owe their existence to. In the analysed remains of the structure, it is difficult to find such procedures, since the construction of the walls was performed on the principle of emplekton masonry, where the strength of the masonry would be determined by the strength of the mortar in addition to the strength of the blocks.

On the other hand, another commonly used procedure was found in the studied structure, which was the use of layers not connected by mortar in the stone foundation. Such a procedure made it possible to reduce the effect of horizontal forces on the higher parts of the structure, thereby minimizing seismic effects on the structure.

In assessing the resilience of structures beyond those mentioned, the overall geometry is equally important. More compact, single-story structures of a regular shape with a well-connected wall system that increases structural rigidity are more capable to resist seismic loads.

Extensive discussions on modelling the functional reconstruction of buildings after an earthquake include [43,44]. The proposed framework can be used both in the probability-based seismic design of new buildings and for the evaluation of existing buildings.

Microstructural studies of the stone material may provide information about the building’s past, such as whether it may have been burned down. Research on the impact of thermal effects on the limestone microstructure indicates that a temperature between 200 °C and 300 °C is critical, at which both macroscopic properties and the macrostructure show significant changes [45]. Similarly, studies on the mechanical parameters of sandstone heated to different temperatures have indicated significant changes in its properties at temperatures between 400 and 600 °C [46]. The lack of samples from rock massifs, which were the source of raw material, made it impossible to conduct comparative studies on this matter.

The deformability parameters obtained in uniaxial and triaxial stress conditions (Table 2 and Table 3) show that the limestones have over twice higher values of Young’s modulus than the sandstones. Under uniaxial compression conditions, limestones are characterised by Young’s modulus values of the order of 80–82 GPa, while sandstones are of the order of 20–30 GPa; under triaxial compression up to 12 MPa of confining pressure, Young’s modulus values increase to the range of 92–94 GPa in limestones and the range of 30–45 GPa in sandstones, respectively. 

**Figure 10:**
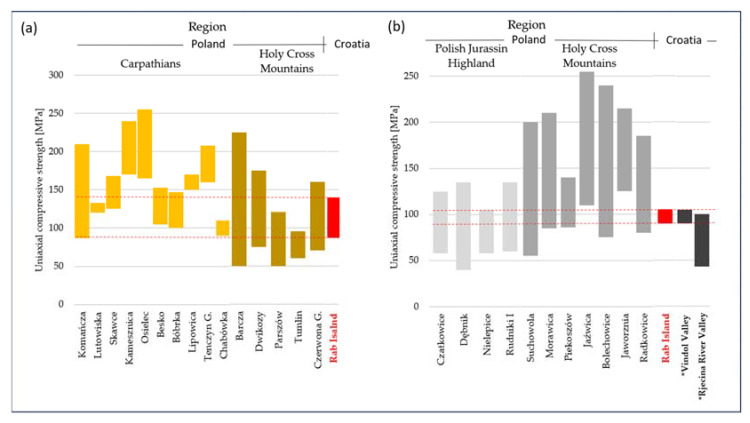
Uniaxial compressive strength of rock tests against the strength of rocks from the region of Poland and Croatia: (**a**) sandstones, (**b**) limestones. *—Limestone USC results from Vindol Valley and Rjecina River Valley based on [47].

The analysis of the endurance values resulting from uniaxial compression indicates that the investigated rocks are characterised by high or very high endurance. Taking into consideration the endurance of sedimentary rocks, subject to the same processes from the area of Poland, their parameters range from 40 to 250 MPa (Figure 10). Thus, the values obtained in this study are in the middle of this range.

The results of uniaxial compressive tests for limestones were also compared with the results of rocks from Croatia from the literature. The range of USC results for the studied Rab Island limestones are very similar to the results of limestones from the area of the western part of Croatia, specifically the Vindol Valley and Rječina River Valley sites [45], except that the Rječina River Valley limestones are more weathered than the Rab Island limestones. The macroscopic comparison of the Rab Island limestones to limestones from Western Croatia indicates that they are closer to the Rječina River Valley limestones, whose strength is lower. The UCS studies reported by Pollak et al. [48] from 11 sites spread mainly around Southern and Western Croatia present a very large range of results (UCS—25–273 MPa, mean 127 MPa). The authors explain this fact by the structural and lithological changes and limestone weathering effects, and they propose an equation to estimate the UCS based on a visual assessment. Following the classifications presented in [47], the limestones from Rab Island are characterised by significantly lower uniaxial compressive strength than the similar ones presented there, and they are closer in strength to the highly fractured, moderately weathered, and moderately porous limestones.

## 6. Conclusions

The first important conclusion of this study is the insights into the process of organising the construction site. It is now clear that mostly local material that met the appropriate strength criteria was largely used to construct the analysed Roman structures.

Although the GPR survey allowed us to determine the almost complete layout of several buildings in Podšilo Bay, their elevation could not be inferred. Results of geomechanical tests presented in this paper are the first step in the determination of the maximum possible height of the analysed walls, which is of fundamental importance to the question of whether the examined building was single-story or higher.

Microstructural observations using microtomography have provided three-dimensional insights into the structure of the rock samples. Using structural reconstruction techniques, the distribution of porosity was evaluated, and the presence of voids or discontinuities was identified. Particularly, the high utility of this type of study was confirmed in the case of limestone samples, where the course of secondarily filled fractures was visualised. Conducting density distribution analyses allows for a preliminary differentiation of the rock material from the fracture fill material. This is of crucial importance during strength assessment. Imaging the course of discontinuities in the analysed material can be helpful both in the selection of test samples with a larger number or in the case of a shortage of test materials, assisting in the interpretation of test results. The performed microtomographic imaging is compared with post-failure testing.

The study of mechanical parameters shows that limestones have a slightly higher strength and four-times-higher Young’s modulus than sandstones. Compressive strengths of both types of rocks compared to typical building materials such as concrete in the most commonly used classes C35–C50, or building ceramic materials, are several times higher. This makes it clear that the selected stone building materials were appropriate for the construction of such structures.

Archaeological implications of the obtained results are twofold. Firstly, the use of locally available sandstone was confirmed, while the source of limestone should be sought in other areas of the island or its adjacency. Secondly, the use of different types of rock—one locally sourced and one transported to the construction site from afar—might be indicative of the builders’ know-how related to the available construction materials, that is, of their inherent properties and suitability. The selection of a locally available sandstone, a practice rarely seen in the coastal area of the Dalmatia province, which, despite being less strong than the tested limestone is still an acceptable construction material, is indicative of the optimisation of construction material sourcing and might signal an economic rationale behind this choice. This would also imply that the Roman builders continued to adapt to the resources available close to the building site, which, in this case, is further supported by the on-site production of tiles. Builders, despite the lack of access to contemporary research methods, had the necessary know-how to adequately select the material in terms of its endurance.

The presented results of research on stone blocks from Rab Island combined with assessments of the building substrate will allow, in the future, us to conduct modelling and reconstruction of the walls of the damaged buildings. This might shed light on the original appearance of the building, which is of fundamental importance for a better understanding of the original function and utilisation of the structures.

## Figures and Tables

**Table 1:** Description of microstructures visible in microtomography.

Lithological Type	Structural Characteristics Based on Computed Microtomography Test Results
LS_1 fine-grained sandstone	Compact structure, no cracks with a dilation exceeding ~0.04 mm. CT porosity (0.02%) very low at a given voxel of 41.5 um. Equivalent pore diameter dominating in the smallest range of 41.5–124.5 μm (98% contribution to porosity).
LS_3 medium-grained sandstone	Compact structure, no cracks with a dilation exceeding ~0.05 mm. Image of pore space, characterised by fine gradation of pores in the test sample. Recorded CT porosity of sample: ~0.08%, where pores of about 0.1 mm (about 74%) dominate; voxel size 46.5 μm.
LS_6 fine-grained sandstone	Grain size about 0.2 mm, CT porosity low (0.17%), pores characterised mainly by an equivalent diameter of 41.5—207.5 μm (80%). Structure compact, with single larger voids or cracks.
LL_7 mudstone	Compact structure, individual pores larger than voxel size (46.5 μm), some cracks filled with secondary mineralization (presence of characteristic “seam” forms).
LL_10 sparitic limestone	Compact structure, with small pores and individual cracks filled with secondary mineralization.

**Table 2:** Identification properties of the analysed lithological types.

Lithological Type		Colour	Bulk Density ρ [kg/m^3^]	Longitudinal Wave Velocity V_p_ [m/s]
			$\underline{x}$ σ (N)	
Sandstone	LS_1	dark grey	2609 17 (11)	4632 248 (17)
	LS_3	light brown	2541 24 (12)	3258 232 (17)
	LS_6	light grey	2561 37 (12)	3434 255 (12)
Limestone	LL_7	dark grey	2637 40 (12)	6335 135 (18)
	LL_10	grey	2664 44 (10)	6375 124 (18)

$\underline{x}$—average, σ—standard deviation, (N)—population size.

**Table 3:** Uniaxial compression and tensile test results.

Lab. Symbols	Indirect Tensile Strength σ_t_ [MPa]	Uniaxial Compression Tests		
		Uniaxial Compressive Strength σ_u_ [MPa]	Young’s Modulus E [GPa]	Poisson’s Ratio ν [-]
	Min-Max $\underline{x}$			
LS_1	11.5–14.5 (13.5)	129	30	
LS_3	5.8–8.0 (7.0)	87–91	17–20	0.23
LS_6	5.5–80 (6.5)	140	27	
LL_7	4.5–9.9 (7.6)	105	90	
LL_10	9.8–117 (10.6)	90	82	

Min—minimum, max—maximum, $\underline{x}$—average.

## Data Availability

Data are contained within the article and Supplementary Materials.

## Supplementary Materials

The following supporting information can be downloaded at: https://www.mdpi.com/article/10.3390/ma17020359/s1, Results of EDS analysis for sandstones and limestones from the site at Podšilo Bay in Lopar.
